# Computational structure prediction of lanthipeptides with NMR data reveals underappreciated peptide flexibility

**DOI:** 10.1002/pro.70252

**Published:** 2025-08-18

**Authors:** Claiborne W. Tydings, Jens Meiler, Allison S. Walker

**Affiliations:** ^1^ Department of Chemistry, Center for Structural Biology, Institute of Chemical Biology Vanderbilt University Nashville Tennessee USA; ^2^ Department of Pharmacology, Center for Structural Biology, Institute of Chemical Biology, Center for Applied Artificial Intelligence in Protein Dynamics Vanderbilt University Nashville Tennessee USA; ^3^ Institute for Drug Discovery, Faculty of Medicine, Faculty of Mathematics and Informatics, Faculty of Chemistry and Mineralogy Leipzig University Medical School Leipzig Germany; ^4^ Center for Scalable Data Analytics and Artificial Intelligence ScaDS.AI and School of Embedded Composite Artificial Intelligence SECAI Dresden Germany; ^5^ Department of Biological Sciences Vanderbilt University Nashville Tennessee USA

**Keywords:** conformation analysis, conjugation, cyclic peptide, ensemble, flexible, lanthipeptide, NMR, non‐canonical amino acid, Rosetta, sidechain, structure prediction

## Abstract

Lanthipeptides are a class of thioether‐containing ribosomally synthesized and post‐translationally modified peptides, which often have antibiotic activity. As a potential starting point for therapeutics, interest in engineering lanthipeptides is growing. Our inability to computationally model and design lanthipeptides in molecular modeling and design software such as Rosetta limits our ability to rationally design lanthipeptides for drug discovery campaigns. We propose that implementing support for the lanthionine rings and dehydrated amino acids found in lanthipeptides will enable accurate lanthipeptide modeling with Rosetta. We find that when compared to the ensembles of lanthipeptides with NMR‐determined structures in the PDB, lanthipeptide ensembles generated with Rosetta have similar experimental agreement, lower Rosetta energy scores, and greater flexibility. Our use of ensemble‐averaged NOE distances instead of requiring individual structures to satisfy all NOE restraints was key for revealing the flexibility of these peptides. Our Rosetta lanthipeptide ensembles show increased flexibility in non‐cyclized peptide regions as well as increased lanthionine ring flexibility when internal hydrogen bonds are absent and glycine residues are present. Support for lanthipeptides in Rosetta enables the design and modeling of lanthipeptides in Rosetta for therapeutic development.

## 1 INTRODUCTION

**FIGURE 1:**
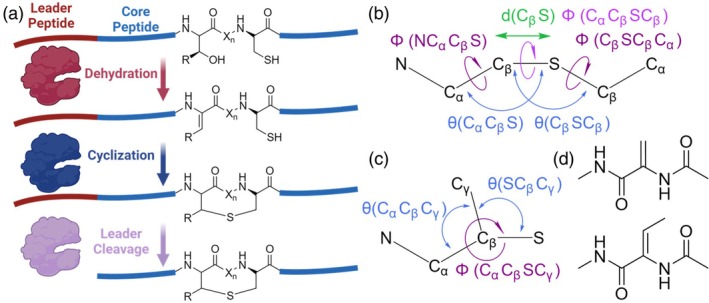
Lanthipeptides are ribosomally synthesized and post‐translationally modified peptides. (a) Lanthipeptides contain a leader peptide for enzymatic recognition and a core peptide that is modified. Serine and threonine residues are dehydrated in lanthipeptides and serve as Michael acceptors for nucleophilic attack by cysteine residues to form lanthionine rings. (b) For lanthionine rings, the important parameters for modeling the cyclization are the new bond distance and the two angles and three dihedrals involving this bond. (c) For methyllanthionine rings, the addition of bond angle terms and an improper dihedral term involving the methyl groups helps to maintain the proper geometry. (d) The dehydrated amino acids are often seen in the final lanthipeptide product and are important to model. Shown here are dehydroalanine (top) and dehydrobutyrine (bottom). Created in BioRender. Tydings, C. (2025) https://BioRender.com/uoltx29

Antibiotic resistance is a global health problem that contributes to an increase in the number and mortality of bacterial infections (Martens & Demain, 2017). To combat bacterial infections, natural products have long been the source of new antibiotics. For example, nisin is a lanthionine ring‐containing ribosomally synthesized and post‐translationally modified peptide (RiPP) with antibiotic properties that has been used in the food industry since 1953 (Shin et al., 2016). Nisin and other RiPPs with lanthionine rings are called lanthipeptides. The properties of lanthipeptides are tuned by their enzymatically introduced thioether sidechain conjugations, called lanthionine rings, that conformationally constrain the lanthipeptides and confer proteolytic resistance (Hegemann et al., 2019). These rings are formed via dehydration of a serine or threonine residue and subsequent nucleophilic attack of the dehydrated amino acid in a Michael addition reaction. Both the lanthionine rings and the dehydrated amino acids can appear in the final peptide product (Arnison et al., 2013) (Figure 1). In attempts to gain insights into the mechanisms of lanthipeptide biological activity, the structures of several lanthipeptides in solution have been determined, primarily with NMR spectroscopy (Acedo et al., 2019; Bobeica et al., 2020; Pei et al., 2022; Shenkarev et al., 2010; Zimmermann & Jung, 1997). These structures revealed that lanthipeptides have diverse conformations with either large alpha helical segments or a lack of traditional secondary structure. Additionally, lanthipeptides have been applied in bioengineering, scaffolding, and library screening (Hegemann et al., 2019; Hetrick et al., 2018; Schmitt et al., 2019; Urban et al., 2017). However, these approaches have not utilized computational modeling of lanthipeptides to inform their engineering strategies.

Currently, many molecular modeling and design software applications such as Rosetta (Leman et al., 2020) cannot model lanthipeptides. Implementation of lanthipeptide modeling in Rosetta would enhance our abilities to both predict the structures of lanthipeptides and perform structure‐based design of lanthipeptides. To enable lanthipeptide modeling in Rosetta, we implement lanthipeptide parameters for lanthionine rings and dehydrated amino acids in the Rosetta codebase. We benchmark Rosetta lanthipeptide structure prediction on 10 lanthipeptides with NMR‐determined structures in the PDB. We find that when compared to lanthipeptide ensembles in the PDB, Rosetta lanthipeptide ensembles refined with NMR data have similar agreement with experimental data, lower Rosetta energies, and higher flexibility. The higher lanthipeptide flexibility is a result of using ensemble‐averaged NOE distances when fitting a lanthipeptide ensemble instead of requiring individual structures to satisfy all NOE restraints. The Rosetta lanthipeptide ensembles show increased flexibility in non‐cyclized peptide regions as well as increased lanthionine ring flexibility when internal hydrogen bonds are absent, and glycine residues are present. The lower Rosetta energy scores indicate that the Rosetta‐predicted ensembles likely have lower free energy than ensembles deposited in the PDB and that lanthipeptides are more flexible than existing ensembles suggest.

## 2 RESULTS

### 2.1 Lanthipeptide parameterization

**FIGURE 2:**
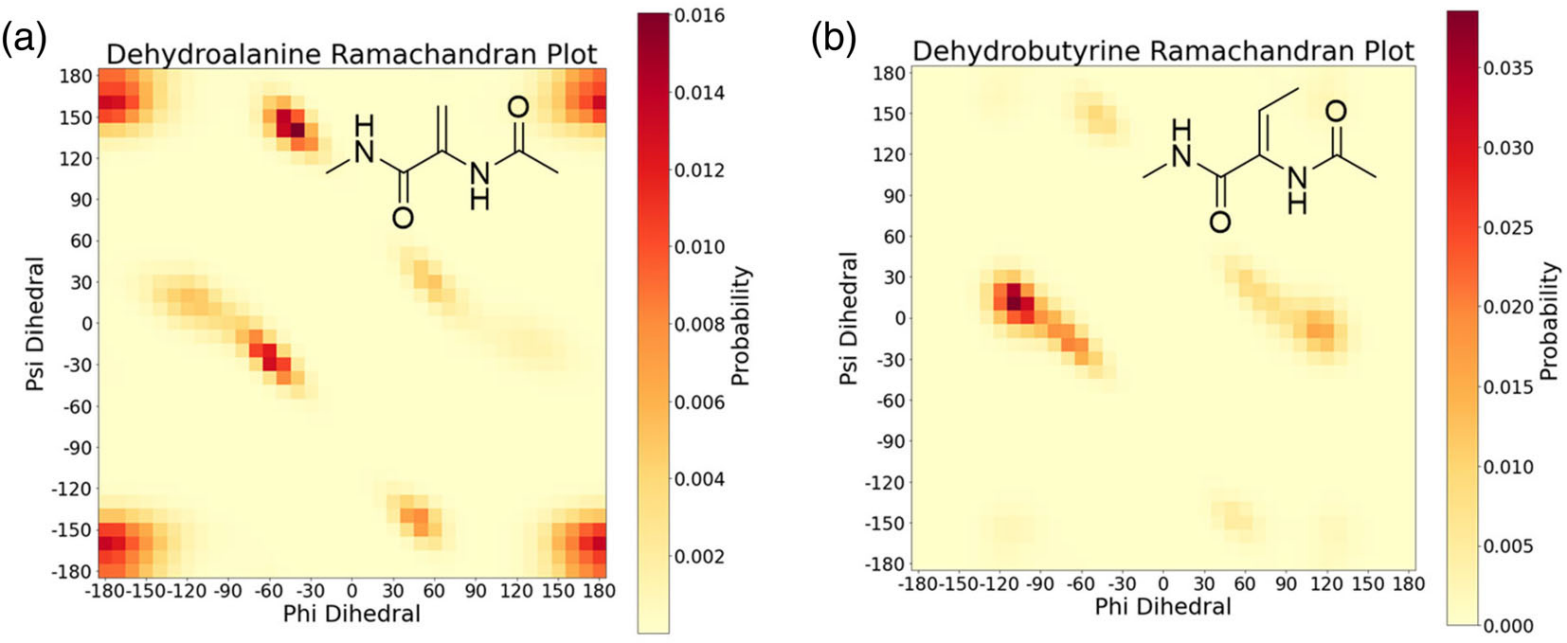
Dehydrated amino acids Ramachandran plots were generated in a 2D scan with the M05‐2X/6‐311g(d,p) functional in the context of neighboring alanine residues. (a) The Ramachandran plot for dehydroalanine. (b) The Ramachandran plot for dehydrobutyrine.

To accurately model the lanthionine ring sidechain conjugations and the dehydrated amino acids found in lanthipeptides, the introduction of new force field terms is required. For sidechain conjugations such as a lanthionine ring, these terms include the distance of the new C–S bond, two angles adjacent to the bond, and the three dihedrals defining the bond's conformation (Alford et al., 2017). Parameters were taken from the Amber (Tian et al., 2020) protein sidechain potentials for the torsion angles and Rosetta cartesian terms (Alford et al., 2017) for the bond distances and angles. For methyllanthionine, an improper dihedral term and additional angle terms involving the methyl group aided in the accurate modeling of its geometry (Figure 1). To model the dehydrated amino acids found in lanthipeptides, new Ramachandran tables were generated. The M05‐2X/6‐311G(d,p) functional was used to perform a 2D phi and psi angle scan for dehydroalanine and dehydrobutyrine (Figure 2) because that functional has previously been shown to accurately capture the torsional potentials of protein backbones (Tian et al., 2020). The nature of the sp^2^ alpha carbon for dehydroalanine and dehydrobutyrine amino acids alters their Ramachandran potentials compared to the canonical amino acids. Additionally, the Ramachandran potential of dehydrobutyrine is further restrained to avoid steric clashes between the gamma carbon and the backbone. After implementing these parameters in Rosetta, we benchmarked lanthipeptide structure prediction with Rosetta for 10 lanthipeptides with NMR‐determined structures in the PDB.

### 2.2 Lanthipeptide ensemble generation with Rosetta

**FIGURE 3:**
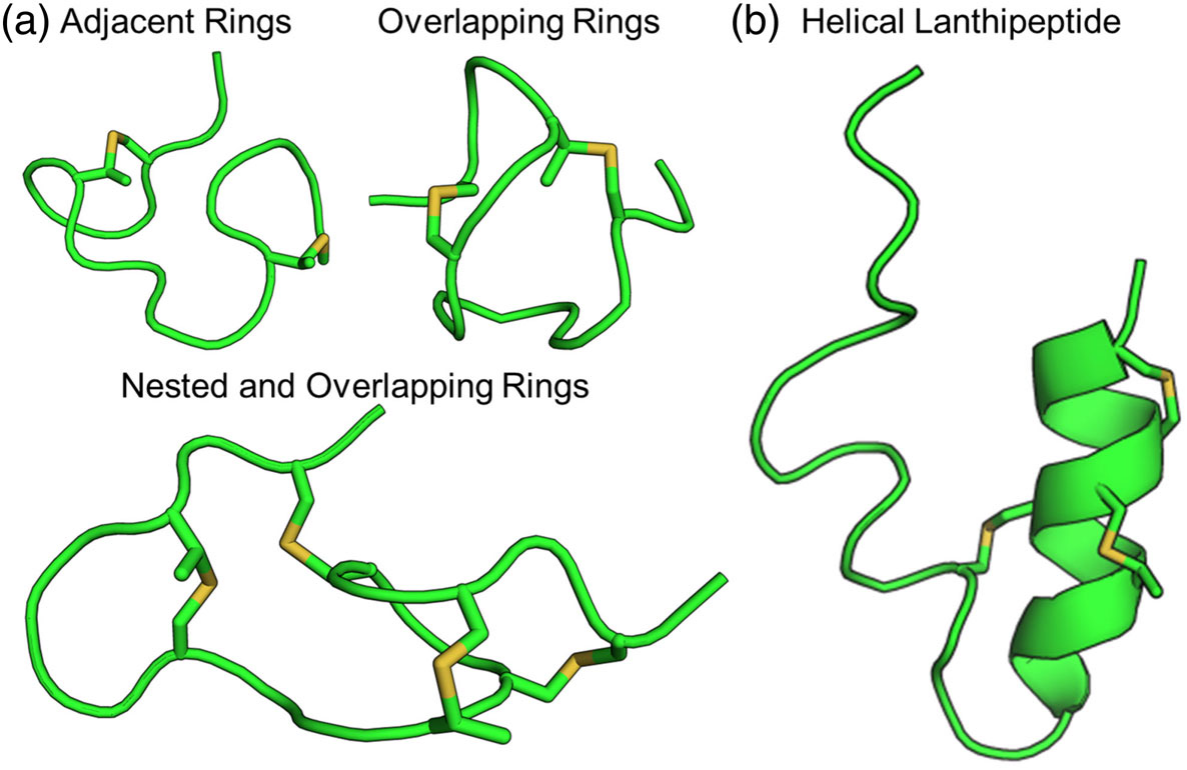
Lanthipeptides can adopt various topologies. (a) Lanthipeptide topologies include adjacent rings, overlapping rings, and nested rings. (b) Some lanthipeptides include large helical segments.

To benchmark the performance of lanthipeptide structure prediction with Rosetta, we first used Rosetta to predict the structures of 10 lanthipeptides with NMR‐determined structures in the PDB. Helical and non‐helical lanthipeptides have quite different structures. Thus, we use different structure prediction protocols for these differently structured peptides. For helical lanthipeptides, lanthionine rings commonly stabilize helices at the ends of helical segments (Figure 3). Specific peptide regions between, on the edges, or within helical segments are flexible. These flexible regions often contain helix breakers such as glycine and proline. The non‐helical peptides have various ring topologies such as adjacent, overlapping, and nested rings and differing numbers and sizes of these rings (Figure 3). The presence of unstructured regions and complex ring topologies in non‐helical lanthipeptides requires far more sampling to explore the larger potential conformational space of the lanthionine rings and the flexible, non‐cyclized peptide regions. Regardless of the structure prediction algorithm, the process of determining an ensemble that satisfies the experimental data is the same and mirrors previously published Monte Carlo protocols to traverse the ensemble landscape guided by experimental data. This protocol is summarized in the methods section (Vortmeier et al., 2015).

**FIGURE 4:**
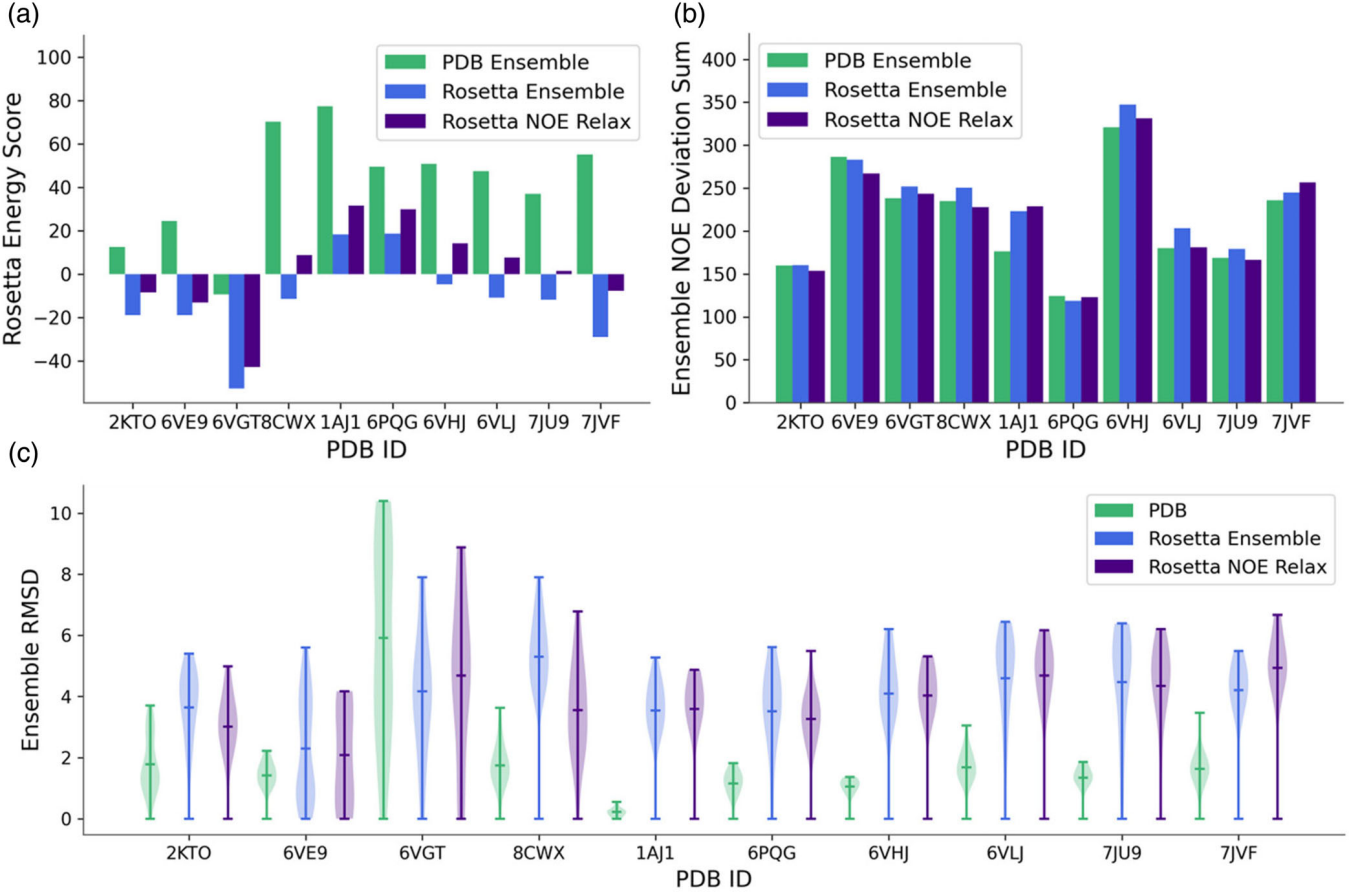
Comparison of global ensemble features between PDB and Rosetta lanthipeptide ensembles. (a) The minimum Rosetta energy score for PDB and Rosetta ensemble members. (b) The ensemble NOE agreement of PDB and Rosetta ensembles. (c) The backbone RMSD to the lowest scoring ensemble member for PDB and Rosetta ensembles.

After using Rosetta to predict lanthipeptide structures, we selected an ensemble of lanthipeptide structures based on agreement with experimental NOE data. Instead of requiring each member of the ensemble to satisfy all experimental data, we optimized the lanthipeptide ensemble to satisfy ensemble averaged distance restraints by using an ensemble averaged NOE distance (Equation 1) (Ashkinadze et al., 2022). Compared to the ensembles in the PDB, the Rosetta‐generated ensembles have similar agreement with experimental data, higher flexibility, and lower Rosetta energies (Figure 4). The responsible score term for the lower Rosetta energies can vary. The score terms that are commonly, but not always, lower in energy include fa_atr, fa_rep, fa_sol, fa_elec, and rama_prepro (Figure S1). Additionally, as a control, we relaxed Rosetta‐generated lanthipeptide conformations with experimental NOE constraints. From the resulting lanthipeptide conformations, we selected 20 structures that agreed well with the NOE constraints and had low Rosetta energy scores as our control ensemble. For this ensemble, we see similar agreement with experimental data, higher flexibility, and lower Rosetta energies compared to the PDB ensemble. However, optimization to the ensemble averaged distance constraints resulted in lower Rosetta energies (Figure 4). Overall, these data indicate that by optimizing an ensemble to satisfy experimental data instead of requiring all structures to individually satisfy those data, we can identify multiple low energy conformations that altogether satisfy the experimental data.

(1) $$d_{\alpha \beta }^{*}={\left(\frac{1}{N}\sum_{i=1}^{N}{\left(d_{\alpha \beta }^{i}\right)}^{-6}\right)}^{-\frac{1}{6}}$$

$d_{\alpha \beta }^{*}$ is the ensemble averaged distance where *N* is the number of equally populated protein states and $d_{\alpha \beta }^{i}$ is the distance between atoms α and β in state *i*.

### 2.3 Structure prediction of helical lanthipeptides

**FIGURE 5:**
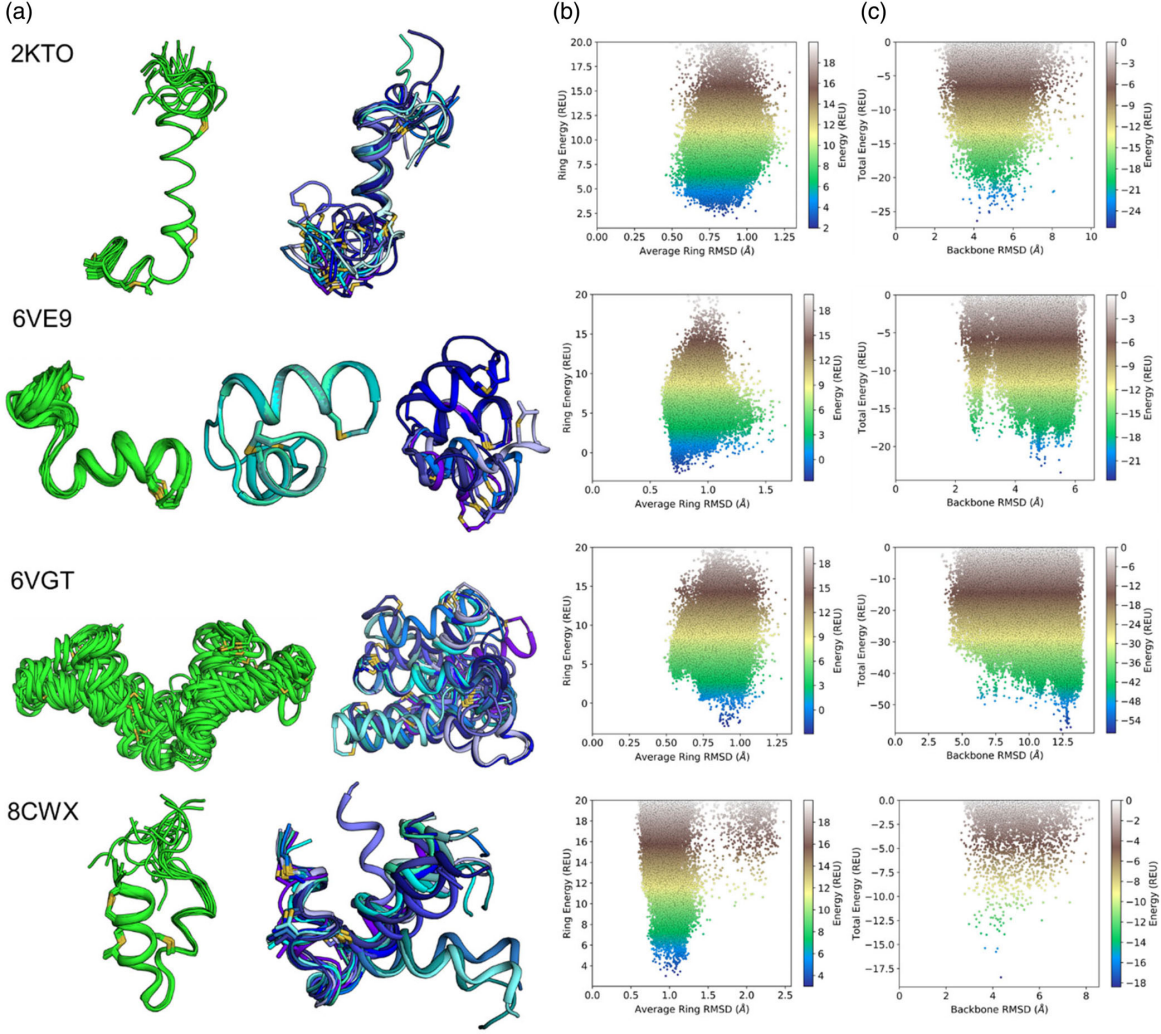
Comparison of PDB and Rosetta ensembles for four helical lanthipeptides. (a) In green is the PDB ensemble and in blue is the ensemble generated with Rosetta. (b) For all Rosetta sampled peptide conformations, the average lanthionine ring RMSD compared to the PDB conformation is plotted on the *x* axis and the Rosetta energy of the rings is plotted on the *y* axis. (c) For all Rosetta sampled peptide conformations, the backbone RMSD compared to the PDB conformation is plotted on the *x* axis and the total Rosetta energy is plotted on the *y* axis.

We used Rosetta to predict the structure of four lanthipeptides with large helical portions. We found that canonicalized AlphaFold2 (Jumper et al., 2021) models of these peptides provided excellent starting points for modeling these peptides, as AlphaFold can successfully identify the helical nature of these peptides. We then used Rosetta to form the lanthionine rings, add the dehydrated amino acids, and sample different lanthipeptide conformations. For these four helical lanthipeptides, sampled lanthionine ring conformations primarily deviated one angstrom or less from the conformation of the lanthionine ring in the PDB. For the global structures of these lanthipeptides, peptide conformations that deviated by up to 6 Å or more from the conformation in the PDB (Figure 5). In contrast, the structures within the PDB ensemble deviate by 2 Å or more within the ensemble, and relaxing these PDBs in Rosetta moves their conformation away from the PDB conformation (Figure 4 and S2). The flexibility of these peptides can be seen by comparing the lanthipeptide ensembles in the PDB to the ensembles generated (Figure 5) with Rosetta. Overall, these data show that for helical lanthipeptides, the individual lanthionine rings are rigid due to their small size and their stabilization within alpha helical structures. When predicted with Rosetta, these helical lanthionine rings generally adopt conformations that differ by 1 Å or less compared to the PDB conformation. The global flexibility in these peptides primarily comes from flexible tails or flexible regions between alpha helical segments.

**FIGURE 6:**
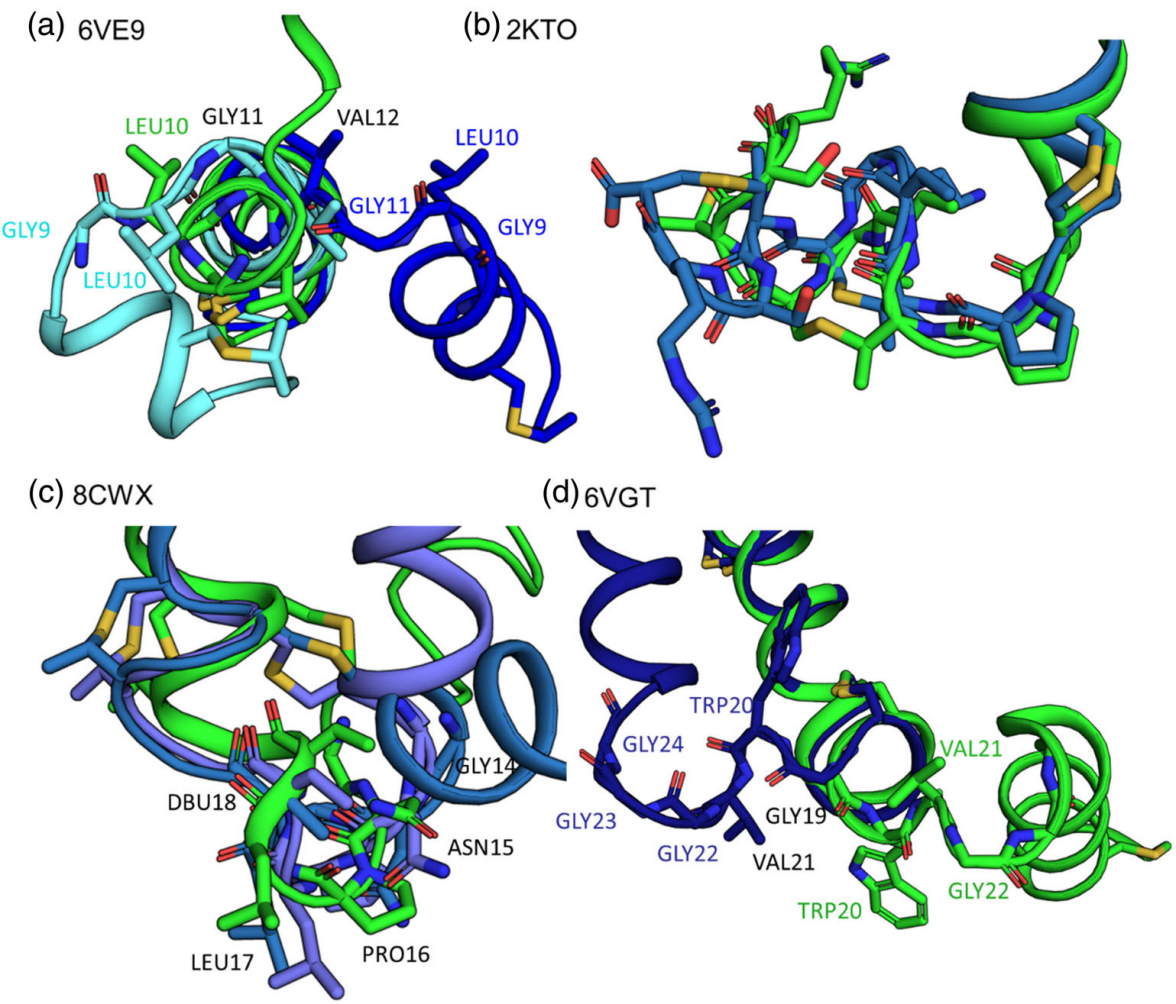
Key components of helical lanthipeptides contribute to their flexibility. (a) Peptide 6VE9 has a segment composed of GLGV. The Rosetta conformations in blue show how the glycine residues in this segment could adopt different conformations than the PDB conformation in green. (b) Peptide 2KTO has two adjacent lanthionine rings at the C terminus. The Rosetta conformation in blue shows how the orientation of these rings could differ from the PDB conformation in green. (c) For peptide 8CWX, the PDB conformation in green and Rosetta conformations in purple are quite similar; however, conformational changes at between residues 14 to 18 in the Rosetta conformation enable large changes in the orientation of the N and C termini compared to one another. (d) For peptide 6VGT, both the Rosetta conformation in blue and the PDB conformation in green show how a segment of three glycine residues and their neighbors introduce flexibility into this peptide.

Helical lanthipeptides have lanthionine rings that stabilize certain helical or non‐helical segments as well as flexible regions that contribute to global flexibility. For example, in peptides 6VGT and 6VE9, several glycine residues introduce flexibility into helical structures. For 6VE9, three glycine residues in the center of the peptide introduce flexibility. In the PDB ensemble, two glycines are helical and one is at the edge of a loop region. This loop is part of a helix bend that allows PHE6 and LEU15 to interact as is seen in the NOE data. However, this data conflicts with backbone chemical shift data and TALOS‐N (Shen & Bax, 2013) predictions which suggest the full peptide is mostly helical (Table 1). In contrast to the PDB ensemble, the Rosetta ensemble has two main conformations with non‐helical regions between residues 7 and 10 or residues 10 and 13 (Figure 6). Though, LEU10 is sometimes helical. Altogether, these data indicate that allowing diverse conformation in the ensemble for 6VE9 enables it to satisfy both the NOE data and helical nature of the peptide. For 6VGT, a segment of three adjacent glycine residues and a fourth glycine another three residues away enable flexibility between two large helical segments in both the PDB and Rosetta ensembles (Figure 6). For PDB 8CWX, a lanthionine ring links the two helical regions and changes in the conformation of that ring, primarily mediated by multiple residues, alter the interactions between those two helical segments in the Rosetta ensemble (Figure 6). For 2KTO, the PDB and Rosetta ensembles are quite similar, but the Rosetta ensemble is more flexible primarily due to perturbations in the linkers between adjacent lanthionine rings at peptide C terminus (Figure 6).

**TABLE 1:** Secondary structure prediction for peptide 6VE9 with TALOS‐N.

XBPACFBIGLGVGALFJAKFC
‐‐‐cH‐‐‐HHHHHHH‐‐‐HHL

*Note*: The sequence of 6VE9 is shown with the secondary structure prediction for each amino acid below. B, J, and X are used to represent dehydrobutyrine, d‐alanine, L‐2‐aminobutanoic acid (methyllanthionine), respectively. The predictions for non‐canonical amino acids and their neighbors are hidden, as these predictions are expected to be inaccurate. “H” is for helical prediction. “L” is for unstructured positions based on chemical shift. “c” is for unstructured positions based on sequence.

### 2.4 Structure prediction of non‐helical lanthipeptides

We used Rosetta to predict the structure of six lanthipeptides without traditional secondary structure. The prediction protocol involved randomizing the backbone of each peptide based on the Ramachandran map, iteratively closing each lanthionine ring, and relaxing the peptide structure. For these peptides, while lanthionine rings can add conformational restrictions, certain regions of the peptide, such as uncyclized termini or the regions between adjacent rings, introduce large amounts of flexibility into these peptides. For the analyzed peptides, the average sampled backbone ring RMSD deviates between 1 and 3 Å, while the PDB conformation and the sampled global backbone RMSD generally vary from 2 to 8 Å compared to the PDB conformation. In contrast, the structures within the PDB ensemble deviate by 1 to 4 Å or more within the ensemble, and relaxing these PDBs in Rosetta moves their conformation away from the PDB conformation (Figure 4 and S3). These data imply that the lanthionine rings themselves have some conformational rigidity, but the orientations of the rings to one another and non‐cyclized peptide regions introduce flexibility.

**FIGURE 7:**
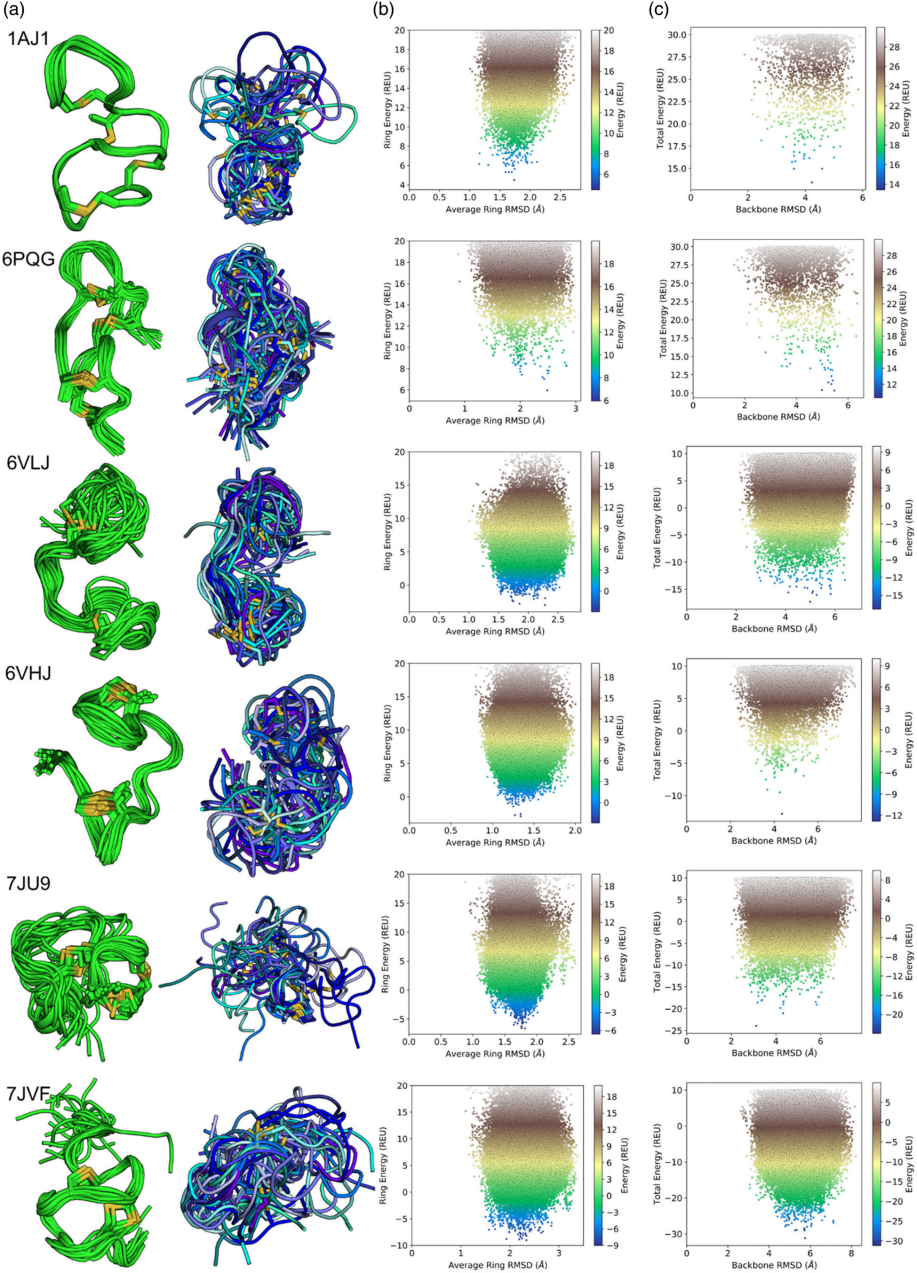
Comparison of PDB and Rosetta ensembles for six non‐helical lanthipeptides. (a) In green is the PDB ensemble and in blue is the ensemble generated with Rosetta. (b) For all Rosetta sampled peptide conformations, the average lanthionine ring RMSD compared to the PDB conformation is plotted on the x axis and the Rosetta energy of the rings is plotted on the y axis. (c) For all Rosetta sampled peptide conformations, the backbone RMSD compared to the PDB conformation is plotted on the x axis and the total Rosetta energy is plotted on the y axis.

For example, the peptides 6VHJ and 6VLJ have two adjacent rings connected by a linker of three or more amino acids. In the PDB ensembles, the orientations of these two rings are relatively consistent; however, the Rosetta ensembles indicate that because no bond holds this orientation in place, these peptides can adopt a range of alternative conformations (Figure 7). In proteins, large structures are held together by well‐packed hydrophobic cores, but peptides do not have these hydrophobic cores. For example, 6VLJ has a sandwich‐like structure in the PDB but no strong interactions hold it in this conformation (Figure 7). Other peptides, such as 1AJ1 and 7JU9, contain non‐conjugated lanthionine rings as well. Like other peptides, the Rosetta ensembles show the orientations of these rings to the rest of the peptide to be more flexible than the PDB ensemble suggests. In contrast, the regions of these peptides that are conformationally constrained by overlapping rings are more conformationally rigid (Figure 7). However, conjugated rings do not automatically make lanthipeptides highly rigid. For example, 7JVF has two overlapping rings and has the greatest sampled average RMSD for its rings (Figure 7). Finally, despite only having connected rings, three overlapping and one nested, 6PQG is more flexible in the Rosetta ensemble than the PDB ensemble (Figure 7). This data indicates that cyclizing a peptide with lanthionine rings reduces peptide flexibility, but cyclization alone fails to rigidify the peptide's conformation.

**FIGURE 8:**
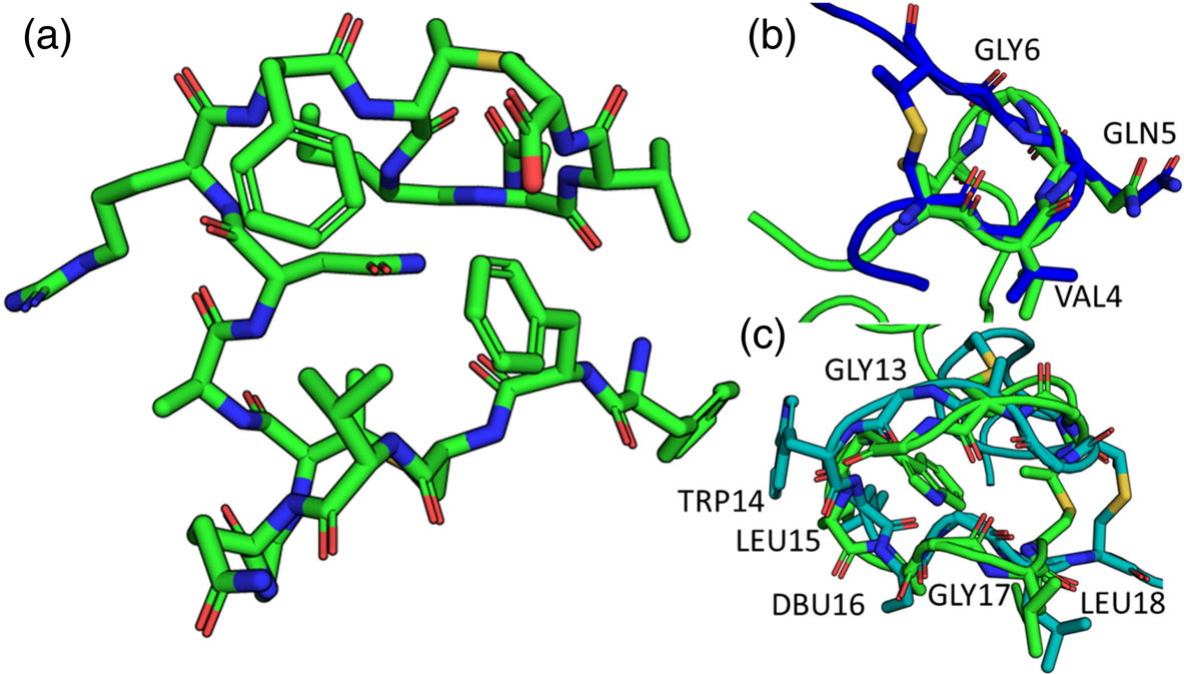
Key components of non‐helical lanthipeptides contribute to their flexibility. (a) Peptide 6VLJ has two lanthionine rings and lacks a stabilizing, well‐packed hydrophobic core. (b) One ring in peptide 6VLJ contains a glycine residue that enables conformational flexibility in structures predicted with Rosetta in blue when compared to the PDB conformation in green. This ring lacks any stabilizing internal hydrogen bonds. (c) A ring in peptide 7JVF has two glycine residues and lacks internal hydrogen bonds. A Rosetta conformation in blue shows conformational changes compared to the PDB conformation in green.

The amino acid composition of lanthionine rings plays a key role in determining the conformational dynamics of those rings. Work to computationally design stable macrocyclic peptides has shown that macrocyclic peptides can be stabilized by proline residues and significant internal hydrogen bonds. Additionally, the conformational preferences of specific amino acids are needed to conformationally stabilize specific positions (Hosseinzadeh et al., 2017; Mulligan et al., 2021). The flexible lanthionine rings lack stabilizing internal hydrogen bonds and often contain glycine residues that enable the flexibility of these rings. For example, 6VHJ has a five‐membered ring example with a glycine residue. In this ring, there are no observable hydrogen bonds, and an overlay of the PDB conformation with a Rosetta conformation shows how the glycine residue enables conformational flexibility in this ring (Figure 8). 7JVF, with the most flexible rings in this set of peptides, has no internal hydrogen bonds and multiple glycine residues in its largest ring. The lack of stabilizing elements in this large lanthionine ring enables that ring to adopt different conformations in the structures predicted by Rosetta (Figure 8).

### 2.5 Filtering of lanthipeptide atropisomers

**FIGURE 9:**
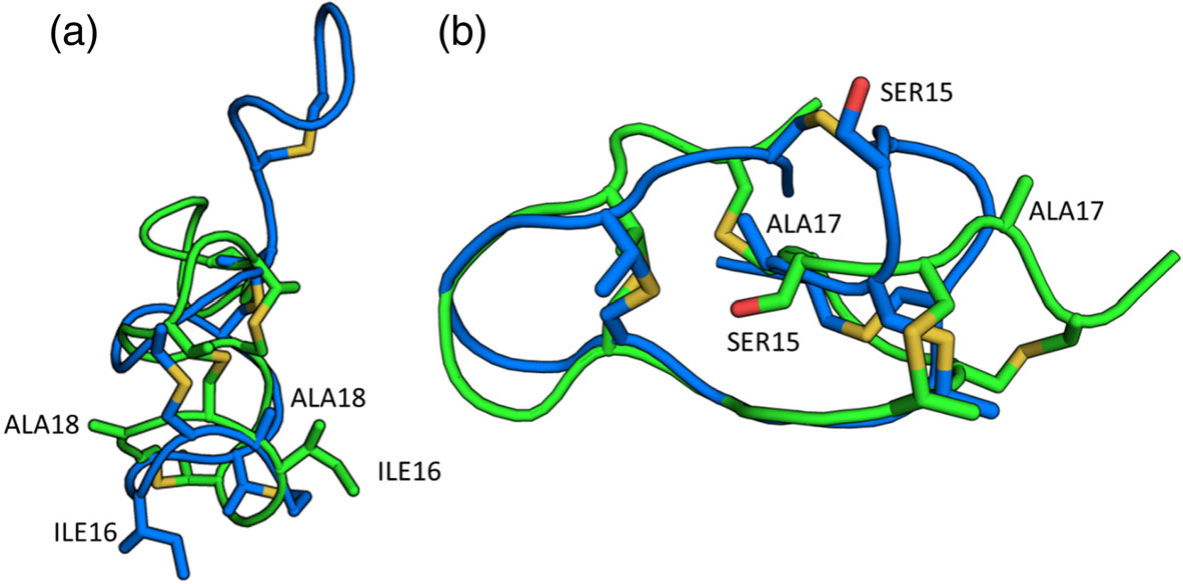
Lanthipeptides with overlapping rings may have non‐interconvertible, atropisomeric conformation. (a) Peptide 1AJ1 has an atropisomer that flips the orientation of the bottom ring. The conformation can be seen by observing how the positions of ILE16 and ALA18 flip with respect to the rest of the peptide. (b) Peptide 6PQG has an atropisomer that flips the orientations of SER15 and ALA17 with respect to the rest of the peptide.

For some unstructured lanthipeptides with overlapping rings, multiple potential atropisomers that cannot interconvert between one another due to steric constraints were observed. These peptides are kinetically trapped similarly to natural products such as lasso peptides and tryptorubin A (Reisberg et al., 2020). For the analyzed lanthipeptides, different atropisomers would sometimes appear in the final ensemble together. The atropisomers were most prevalent for 1AJ1 and 7JU9 (Figure 9). For these peptides, the non‐contributing atropisomers were filtered from the ensemble candidate pool prior to selecting the ensemble. In both cases, the atropisomer observed in the PDB was the majority isomer of the pre‐filtered lanthipeptide structures and the non‐filtered ensembles for those peptides.

## 3 DISCUSSION

Here, we implement parameters for modeling lanthipeptides in Rosetta and use this implementation to predict the structures of 10 lanthipeptides in the PDB with NMR‐determined structures. We find that, compared to the PDB ensembles, using ensemble‐averaged NOE distances to model lanthipeptides in Rosetta leads to ensembles that have similar agreement to the experimental data, lower Rosetta energy scores, and higher flexibility. Several peptide features contribute to their flexibility. Uncyclized regions, either at the termini or between rings, can to adopt a variety of conformations. Within lanthionine rings, the presence of glycine residues and a lack of internal hydrogen bonds enhance the flexibility of those rings. Overall, this data suggests that lanthipeptides are more flexible than PDB ensembles indicate and that peptide conformational dynamics should be considered when determining the structures of lanthipeptides.

Previous studies support our hypothesis that lanthipeptides should be considered in a dynamic structural context. For example, previous study found that cyclization does not automatically rigidify a peptide backbone. Rather, it needs to support the conformational preferences of the cyclized region to result in a rigid macrocycle (Wang et al., 2015). Additionally, for nisin, one of many lanthipeptides that bind lipid II (Repka et al., 2017), the lanthionine rings are not entirely rigid, and the regions connecting lanthionine rings are flexible. Nisin's flexibility enables it to adopt distinct conformations when binding to lipid II and forming membrane pores than it adopts in solution (Dickman et al., 2019; Garcia‐Ruiz et al., 2012). So, in lanthipeptides, flexibility can be important for solubility in water and adopting an active conformation when encountering a target such as a lipid bilayer.

To further improve the modeling of lanthipeptides with experimental data, it would be beneficial to have methods that could accurately and rapidly predict carbon, nitrogen, and hydrogen chemical shifts for non‐canonical amino acids or could predict backbone dihedral angles from experimental chemical shifts. Current methods of chemical shift and backbone dihedral angle prediction such as SHIFTX2 (Han et al., 2011) and TALOS‐N (Shen & Bax, 2013) cannot incorporate non‐canonical amino acids, limiting their utility for large segments of lanthipeptides and the types of experimental data that can be used for lanthipeptide structure determination. As backbone chemical shifts are influenced by the identity of the amino acid and its immediate neighbors (Schwarzinger et al., 2001), predictions for both non‐canonical amino acids and their neighbors are unreliable. Furthermore, current lanthipeptide structure prediction pipelines require prior knowledge of the lanthipeptide ring topology and chiral center configurations.

Our inability to predict the ring topologies of lanthipeptides and the chirality of their lanthionine rings from sequence data alone is a major barrier to de novo lanthipeptide structure prediction. Prediction of these features is a particularly challenging task because the enzyme or the substrate sequence can determine the ring topology and chirality of lanthipeptide substrates (Garg et al., 2016; Le et al., 2021; Sarksian et al., 2022; Tang et al., 2015). For lanthipeptides with nested rings, the structure prediction problem can be compounded by the need to filter atropisomers that do not agree with experimental data.

For peptides that are known to be helical, starting AlphaFold models can reduce the complexity of the structure prediction problem. However, AlphaFold is not a conclusive predictor of peptide helicity (McDonald et al., 2023). For the helical lanthipeptides we examined, we saw certain patterns that supported their helicity. The helical rings in these peptides had a cysteine residue that was three or four residues after the sidechain conjugation partner. For non‐helical lanthipeptides, their ring topologies were often incompatible with helices. Prior to predicting the structure of a lanthipeptide with undetermined structures, we would recommend checking if the ring pattern could support a helical lanthipeptide before using the helical structure prediction pipeline. For small helical peptides, the non‐helical lanthipeptide structure prediction pipeline has potential to accurately predict their structure. A combination of initial random sampling with Monte Carlo‐based fine tuning has previously been used for the structure prediction of cyclic peptides up to 24 amino acids in length without secondary structure constraints or fragments (Zhu et al., 2025). Despite potential for improvement, the methodology for modeling lanthipeptides presented in this study has great potential for identifying new insights into the conformations of these peptides.

In addition to providing a useful method for modeling the conformations of natural lanthipeptides, this study can be applied to engineering new therapeutic lanthipeptides. Current bioengineering approaches involving lanthipeptides rely on screening large libraries of lanthipeptides to find hit compounds (Urban et al., 2017; Yang et al., 2018). Computational modeling and design of lanthipeptides with Rosetta could be used to inform the screening libraries for specific targets or for optimization of hit compounds from these screens.

## 4 CONCLUSION

We implemented the parameters for modeling lanthipeptides in the Rosetta macromolecular modeling software to enable modeling and design of lanthipeptides in Rosetta. We used this implementation to predict the structures of 10 lanthipeptides with NMR‐determined structures in the PDB and show that Rosetta‐generated lanthipeptide ensembles are more flexible than the lanthipeptides in the PDB. These data suggest that lanthipeptides should be considered as dynamic molecules and that lanthionine rings can increase the local rigidity of a peptide but will not necessarily make peptides globally rigid. Methods that can rapidly predict the chemical shifts of non‐canonical amino acids could improve this modeling by refining the ensemble selection process. However, lanthipeptide modeling in Rosetta still provides valuable utility for generating lanthipeptide structural hypotheses, which can deepen our understanding of the mechanism of action of natural lanthipeptides, and as a tool for computationally designing and modeling lanthipeptides with therapeutic potential. These benefits include informing experimental library screens of lanthipeptides and optimizing hit compounds to accelerate the process of lanthipeptide therapeutic development.

## 5 METHODS

### 5.1 Preparation of PDB lanthipeptide ensembles

Prior to energy measurement of lanthipeptide from the PDB ensemble, all structures were relaxed 20 times with backbone restraints in Rosetta. The lanthipeptide Rosetta energy scores were then determined without the backbone constraint term. For RMSD calculations, the backbone RMSD of the unrelaxed PDB structures was computed with respect to the first structure in the PDB ensemble. We used this same structure for RMSD calculations comparing Rosetta‐generated lanthipeptide models to the native structure. For computing model distances of experimentally measured NOE distances, we used the unrelaxed PDB structures.

### 5.2 Helical lanthipeptide prediction pipeline

To predict the structures of lanthipeptides with alpha helical structures, first the lanthipeptide sequences are canonicalized by converting D amino acids to glycine and others to alanine. Additionally, some cysteine residues were mutated to alanine to prevent the formation of disulfide bonds. Using these canonicalized sequences, AlphaFold2 with no templates is used to generate starting models for these peptides. Then, Rosetta is used to mutate the starting models into the true lanthipeptide sequence and cyclize the lanthipeptide. After relaxing the peptides with Rosetta, Rosetta is used to sample lanthipeptide conformations using Monte Carlo‐based fragment insertion, backrub moves, shear moves, and sidechain moves with the XML interface. The lanthipeptide structures are refined with Rosetta relax, and a total of 5000 models are generated for each peptide. We then perform a second round of structure refinement. For each of the 5000 models, we start 20 Monte Carlo trajectories that make small, shear, and sidechain moves. The trajectory involves an initial 1000 burn‐in steps, after which the final pose is passed to another 9000 steps, and the lowest energy structure is recovered and relaxed. The top 1000 of these structures, based on Rosetta energy scores, are used to build the final ensemble.

### 5.3 Unstructured lanthipeptide prediction pipeline

To predict the structures of lanthipeptides without traditional secondary structure, the backbone of these peptides is first randomized based on the Ramachandran map. Then, in the XML interface, generalized kinematic loop closure is used to sample the structure of each lanthionine ring in every possible ring permutation. The lanthipeptide structures are refined with Rosetta relax. 150,000–200,000 peptide structures are generated with Rosetta, and the top 2.5% of these structures, based on Rosetta energy scores, are passed to a second round of structure refinement. We use the same protocol for the second round of refinement as described for the helical lanthipeptide structure prediction. Briefly, this refinement involves 1000 steps of Monte Carlo burn‐in steps and another 9000 steps, after which the lowest energy structure is recovered and relaxed. The top 2.5% of lanthipeptide structures from the second round of refinement, based on Rosetta energy scores, are used to build the final ensemble.

### 5.4 Non canonical amino acid parameterization

Dehydroalanine and dehydrobutyrine were parameterized for Rosetta with the molfile_to_params_polymer.py script included with Rosetta (Bell et al., 2025). To generate the phi and psi Ramachandran potential, a 2D scan of these torsions is performed in Gaussian with the M05‐2X/6‐311g(d,p) functional previously shown to best reproduce the backbone conformational energies for an alanine dipeptide (Tian et al., 2020). The scan of the dehydrated amino acids was performed in the context of adjacent alanine residues. All three residues with n‐methyl and acetyl caps formed a pentapeptide. The lanthionine ring parameters were taken from the Amber (Tian et al., 2020) force field for torsions and the Rosetta cartesian force field for bond distances and angles.

### 5.5 Ensemble generation

The peptide ensemble generation is based on a previously published Monte Carlo protocol for selecting an ensemble that satisfies experimental data (Vortmeier et al., 2015). Briefly, a random set of 20 peptide conformations is selected. This set is perturbed by adding a member, removing a member, or swapping a member with a non‐member so that the ensemble size stays between 10 and 30 members. The sum of the square of the difference between calculated ensemble averaged NOE values and the observed NOE values is used to score each ensemble (Ashkinadze et al., 2022). Moves are accepted or rejected based on a Boltzmann criterion. After 500,000 steps, the lowest scoring ensemble is taken as the final ensemble. If chemical shift data is used, the difference between the SHIFTX2 (Han et al., 2011) predicted chemical shift and the observed chemical shift is added to this score. To ensure that the chemical shift predictions are reliable, chemical shifts are only included for amino acids that are canonical and have canonical neighbors. To compare peptide structures to the PDB ensemble, RMSD calculations were done to the first structure in the PDB ensemble. For the Rosetta ensembles, RMSD calculations were done to the lowest energy structure.

### 5.6 Ensemble generation from models relaxed with NOE restraints

To generate peptide models that are directly informed by experimental NOE measurements, we relaxed all 5000 helical peptide models and the top 2.5% of the non‐helical peptide models for the initial structure prediction round, which is approximately 5000 models, 20 times with NOE measurements as Rosetta constraints. For each of the initial models, we selected the top scoring model, including distance constraints, of the 20 derivative models for the ensemble selection process. To select a final ensemble, we first filtered to the top 200 structures based on agreement with the experimental NOE measurements. We then selected the top 20 models based on Rosetta energy scores as the final ensemble. To compare peptide structures to the PDB ensemble, RMSD calculations were done to the first structure in the PDB ensemble. For the Rosetta ensembles, RMSD calculations were done to the lowest energy structure.

### 5.7 Atropisomer identification

Atropisomers are differentiated by strategically selecting sets of four residue alpha carbons. Three of these carbons are on nearby residues in a loop of interest and form a plane. The fourth carbon is selected to be on a separate loop or region such that it can be determined if this fourth carbon is on the positive or negative side of the vector normal to the plane formed by the other three carbons. Enough groups of four carbons are selected to distinguish all of the potential atropisomers from the atropisomer supported by the NOE data.

### 5.8 Rosetta codebase changes

The changes necessary to model lanthionine rings were added to the crosslinker mover in Rosetta. Additionally, patches were added to Rosetta for the modeling of lanthionine rings and dehydrated amino acids. To enable modeling of NOE distance with non‐canonical amino acids, the AmbiguousNMRDistance constraint in Rosetta was modified to be generalizable. These changes are available in the current version of Rosetta on github at https://github.com/RosettaCommons/rosetta. Rosetta is free to use for academic users and requires a license for commercial users. The code and starting models for the lanthipeptide analysis will be made available at https://github.com/tydingcw/lanthi_folding_protocol_capture.

## AUTHOR CONTRIBUTIONS

**Claiborne W. Tydings:** Conceptualization; investigation; methodology; writing – original draft; validation; visualization; writing – review and editing; software; formal analysis; data curation. **Jens Meiler:** Supervision; writing – review and editing; funding acquisition; conceptualization; resources. **Allison S. Walker:** Supervision; writing – review and editing; funding acquisition; conceptualization; resources.

## Supporting information


**FIGURE S1.** For each lanthipeptide, the minimum Rosetta energy conformation for the PDB ensemble and the ensemble generated with Rosetta and the Monte Carlo selection protocol were chosen. Here, we calculate the difference in Rosetta energy for various Rosetta score terms. Negative energy values indicate that the Rosetta conformer had a lower energy for that score term.
**FIGURE S2.** RMSD vs. energy plots for the helical lanthipeptides. The conformations sampled with the Monte Carlo protocol are shown in small dots. The PDB structures that were relaxed with backbone coordinate constraints are shown in black dots. The PDB structures that were relaxed without any coordinate constraints are shown in red dots.
**FIGURE S3.** RMSD vs. energy plots for the non‐helical lanthipeptides. The conformations sampled with the Monte Carlo protocol are shown in small dots. The PDB structures that were relaxed with backbone coordinate constraints are shown in black dots. The PDB structures that were relaxed without any coordinate constraints are shown in red dots.

## Data Availability

The data that support the findings of this study are available from the corresponding author upon reasonable request.
