# Supplementary material for: Computational structure prediction of lanthipeptides with NMR data reveals underappreciated peptide flexibility
Source: Protein Sci. 2025 Aug 18;34(9):e70252. doi: 10.1002/pro.70252 (PMC12359204; doi:10.1002/pro.70252)
Supplement: Supplementary file 1 — FIGURE S1. For each lanthipeptide, the minimum Rosetta energy conformation for the PDB ensemble and the ensemble generated with Rosetta and the Monte Carlo selection protocol were chosen. Here, we calculate the difference in Rosetta energy for various Rosetta score terms. Negative energy values indicate that the Rosetta conformer had a lower energy for that score term. FIGURE S2. RMSD vs. energy plots for the helical lanthipeptides. The conformations sampled with the Monte Carlo protocol are shown in small dots. The PDB structures that were relaxed with backbone coordinate constraints are shown in black dots. The PDB structures that were relaxed without any coordinate constraints are shown in red dots. FIGURE S3. RMSD vs. energy plots for the non‐helical lanthipeptides. The conformations sampled with the Monte Carlo protocol are shown in small dots. The PDB structures that were relaxed with backbone coordinate constraints are shown in black dots. The PDB structures that were relaxed without any coordinate constraints are shown in red dots. [file PRO-34-e70252-s001.docx]

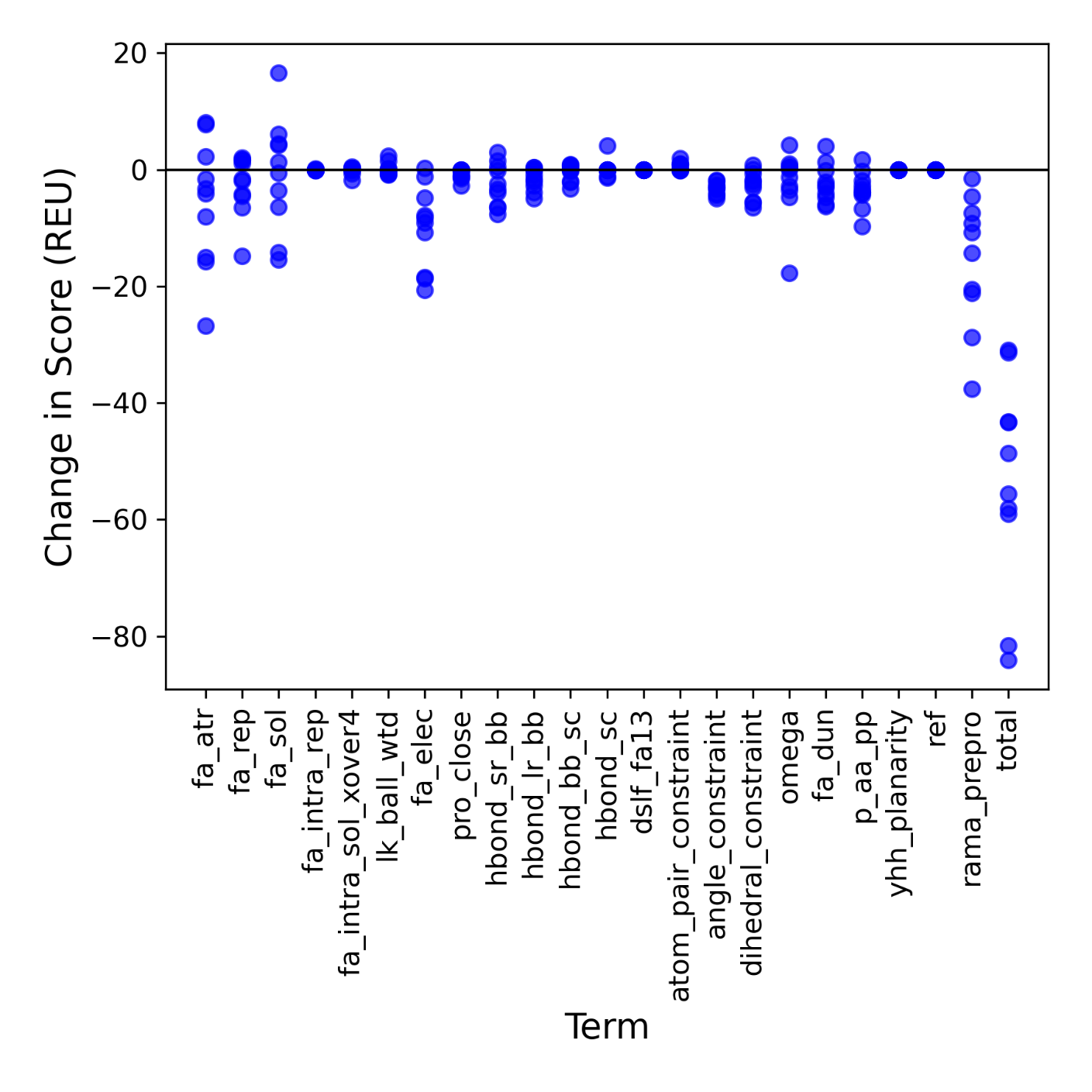


**Figure S1**. For each lanthipeptide, the minimum Rosetta energy conformation for the PDB ensemble and the ensemble generated with Rosetta and the Monte Carlo selection protocol were chosen. Here, we calculate the difference in Rosetta energy for various Rosetta score terms. Negative energy values indicate that the Rosetta conformer had a lower energy for that score term.


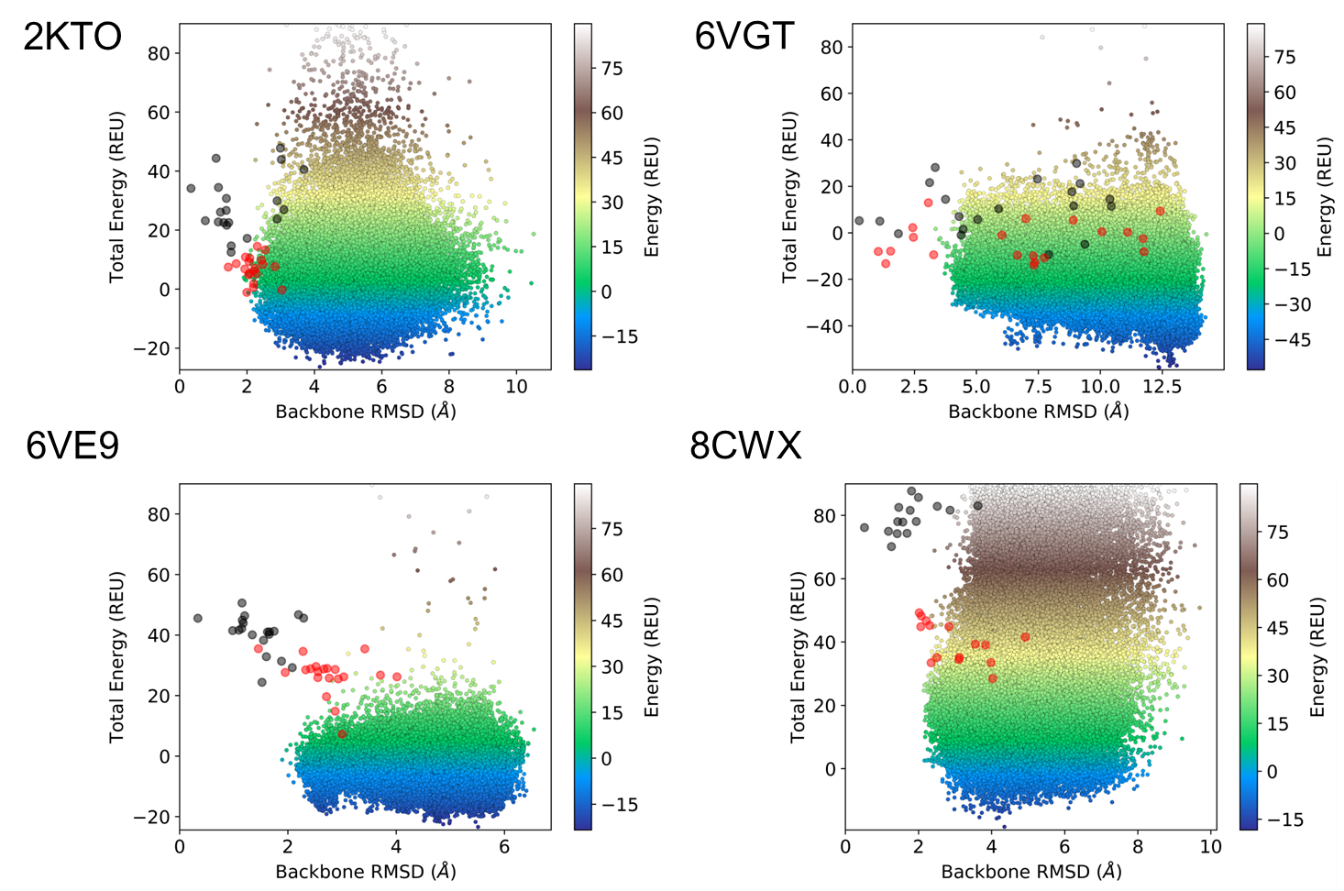


**Figure S2**. RMSD vs energy plots for the helical lanthipeptides. The conformations sampled with the Monte Carlo protocol are shown in small dots. The PDB structures that were relaxed with backbone coordinate constraints are shown in black dots. The PDB structures that were relaxed without any coordinate constraints are shown in red dots


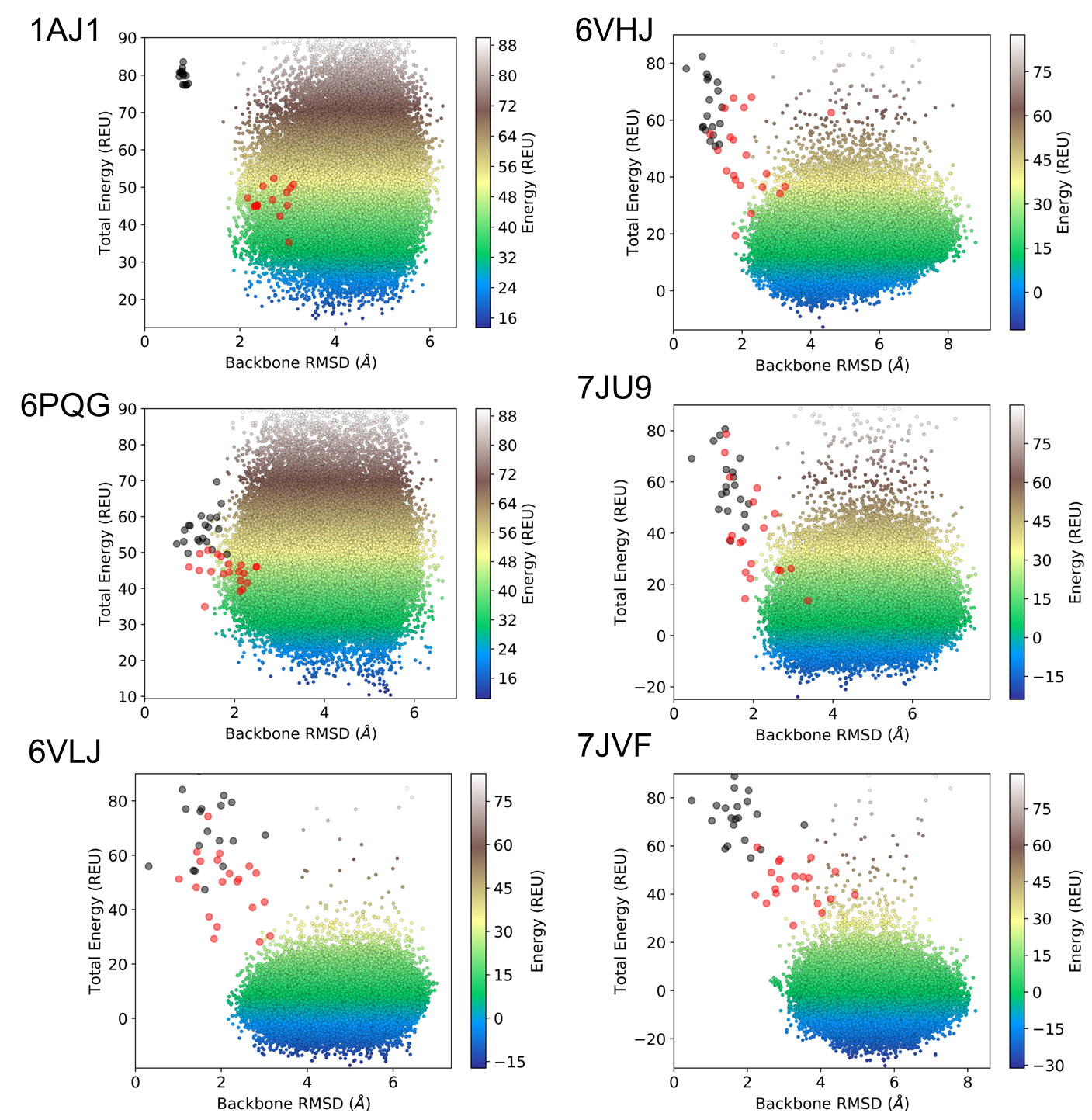


**Figure S3**. RMSD vs energy plots for the non-helical lanthipeptides. The conformations sampled with the Monte Carlo protocol are shown in small dots. The PDB structures that were relaxed with backbone coordinate constraints are shown in black dots. The PDB structures that were relaxed without any coordinate constraints are shown in red dots
